# Impact of harmonization on the reproducibility of MRI radiomic features when using different scanners, acquisition parameters, and image pre-processing techniques: a phantom study

**DOI:** 10.1007/s11517-024-03071-6

**Published:** 2024-03-27

**Authors:** Ghasem Hajianfar, Seyyed Ali Hosseini, Sara Bagherieh, Mehrdad Oveisi, Isaac Shiri, Habib Zaidi

**Affiliations:** 1 Division of Nuclear Medicine and Molecular Imaging, Geneva University Hospital, CH-1211 Geneva, Switzerland; 2 Translational Neuroimaging Laboratory, McGill University Research Centre for Studies in Aging, Douglas Hospital, McGill University, Montréal, Québec Canada; 3 Department of Neurology and Neurosurgery, Faculty of Medicine, McGill University, Montréal, Québec Canada; 4 School of Medicine, Isfahan University of Medical Sciences, Isfahan, Iran; 5 Department of Computer Science, University of British Columbia, Vancouver, BC Canada; 6 Department of Cardiology, Inselspital, Bern University Hospital, University of Bern, Bern, Switzerland; 7 Department of Nuclear Medicine and Molecular Imaging, University of Groningen, University Medical Center Groningen, Groningen, Netherlands; 8 Department of Nuclear Medicine, University of Southern Denmark, Odense, Denmark; 9 University Research and Innovation Center, Óbuda University, Budapest, Hungary

**Keywords:** MRI, Radiomics, Robustness, Pre-processing, Harmonization, Scanner effect

## Abstract

This study investigated the impact of ComBat harmonization on the reproducibility of radiomic features extracted from magnetic resonance images (MRI) acquired on different scanners, using various data acquisition parameters and multiple image pre-processing techniques using a dedicated MRI phantom. Four scanners were used to acquire an MRI of a nonanatomic phantom as part of the TCIA RIDER database. In fast spin-echo inversion recovery (IR) sequences, several inversion durations were employed, including 50, 100, 250, 500, 750, 1000, 1500, 2000, 2500, and 3000 ms. In addition, a 3D fast spoiled gradient recalled echo (FSPGR) sequence was used to investigate several flip angles (FA): 2, 5, 10, 15, 20, 25, and 30 degrees. Nineteen phantom compartments were manually segmented. Different approaches were used to pre-process each image: Bin discretization, Wavelet filter, Laplacian of Gaussian, logarithm, square, square root, and gradient. Overall, 92 first-, second-, and higher-order statistical radiomic features were extracted. ComBat harmonization was also applied to the extracted radiomic features. Finally, the Intraclass Correlation Coefficient (ICC) and Kruskal-Wallis’s (KW) tests were implemented to assess the robustness of radiomic features. The number of non-significant features in the KW test ranged between 0–5 and 29–74 for various scanners, 31–91 and 37–92 for three times tests, 0–33 to 34–90 for FAs, and 3–68 to 65–89 for IRs before and after ComBat harmonization, with different image pre-processing techniques, respectively. The number of features with ICC over 90% ranged between 0–8 and 6–60 for various scanners, 11–75 and 17–80 for three times tests, 3–83 to 9–84 for FAs, and 3–49 to 3–63 for IRs before and after ComBat harmonization, with different image pre-processing techniques, respectively. The use of various scanners, IRs, and FAs has a great impact on radiomic features. However, the majority of scanner-robust features is also robust to IR and FA. Among the effective parameters in MR images, several tests in one scanner have a negligible impact on radiomic features. Different scanners and acquisition parameters using various image pre-processing might affect radiomic features to a large extent. ComBat harmonization might significantly impact the reproducibility of MRI radiomic features.

**Graphical Abstract:**

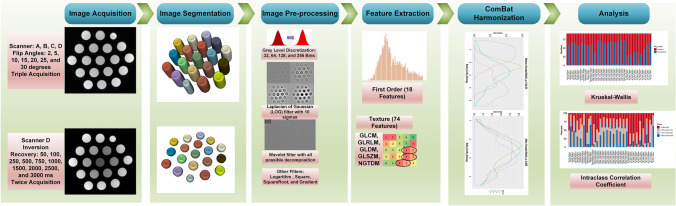

**Supplementary Information:**

The online version contains supplementary material available at 10.1007/s11517-024-03071-6.

## Introduction

Magnetic resonance imaging (MRI) provides detailed, clinically relevant images of soft tissue structures, which are impossible to attain via any other non-invasive medical imaging modality [1–4]. Therefore, it is frequently utilized for cancer diagnosis, staging, and follow-up. However, contrary to positron emission tomography (PET) and computed tomography (CT), which provide images coded in Hounsfield and kBq/mL units, MRI intensity gray levels do not use a particular standard unit owing to the lack of intensity for each specific tissue [1–4]**.** Consequently, the intensity varies for the same MRI scanner, imaging protocol, and biological tissue, hence the necessity of intensity normalization [1–4]. Moreover, the radiomic feature reproducibility extracted from MR images is affected by numerous parameters, including but not limited to magnetic field strength, gradient strength, MR sequence, image acquisition protocol, and reconstruction algorithm [1–4].

In combination with machine learning, extracting high-throughput quantitative measures from medical images, referred to as radiomics, is used to create models for prediction, screening, diagnosis, response to treatment, and prognosis using medical images and clinical data [1, 3–5]. Hence, it is essential that radiomic features from various modalities, such as PET [6], CT [7], and MRI [8], be reproducible. In other words, it is crucial to obtain features that can be verified by subsequent research with an identical technique, dataset, and/or patient cohort to confirm that the analysis has been conducted without errors.

Previous studies demonstrated that various factors might impact radiomic features in MR images to a large extent, including image pre-, post-processing [9, 10], test-retest [11], and multi-center [12, 13]. To overcome the low reproducibility of radiomic features, several methods have been proposed, among which selecting reproducible features and ComBat harmonization against influential factors seem to be plausible solutions [14].

Harmonization approaches were developed to improve the repeatability of research on radiomic features using medical imaging by removing undesired impacts of vendor-dependent features or resolving inconsistencies across medical images [15]. Harmonizing MR images is feasible using two distinct approaches, namely prior to and following feature extraction [16, 17]. The present study focuses on the second approach, i.e., using harmonized radiomic features once they have been extracted [16, 17].

ComBat harmonization has been widely used for different imaging modalities in a variety of scenarios, thus demonstrating its ability to decrease radiomic feature variability in CT [18], PET [6], and MRI [19]. This popular method was introduced by Johnson et al. [20] to remove batch effects impacts in microarray expression and then applied to PET, CT, and MR images [6, 18, 21, 22]. In addition, Orlhac et al. [1] used ComBat to eliminate the variability of MRI radiomic features in a multi-center study. Moreover, in another study, Li et al. [3] used this method for harmonized MRI radiomic features extracted from 3 and 1.5 Tesla magnetic field strength scanners.

The variability of radiomic features might be caused by several factors, such as varying flip angles (FAs) and inversion recovery (IR) in the same MR scanner and separate scanners with almost the same protocols and situations (in a phantom study) [23–25]. In MRI, the FA affects signal intensity and contrast in various tissues [23]. While a smaller flip angle speeds up scanning and improves the signal-to-noise ratio, it might impair T1 contrast and saturation recovery [23]. IR determines which tissue will be without signal or nulled according to the selection of inversion time (TI) [24, 25]. Since only one texture is used in the phantom, using different TIs affects signal intensity. The signal from the phantom will be nullified if TI is equal to T1 [24, 25]. The signal from the phantom will be positive or negative depending on whether the TI is shorter or longer than the T1 of the phantom, respectively [24, 25]. A variety of image pre-processing techniques had an additional effect on this variability [9, 10]. The current study aims to investigate the effect of the ComBat harmonization method on the reproducibility of MRI radiomic features for different scanners and acquisition parameters with different image pre-processing techniques using a dedicated MRI phantom study.

The novelty and main contribution of the current study can be summarized in the following items:

- Exploring the effect of ComBat harmonization on the reproducibility of MRI radiomic features;
- Investigating a broad range of inversion recovery (IR) sequences and flip angles (FA) in a nonanatomic phantom from the TCIA RIDER for MRI scans;
- Employing various pre-processing techniques, such as bin discretization, wavelet filters, and Laplacian of Gaussian, to comprehensively evaluate the impact of these methods on the robustness and consistency of MRI radiomic features;
- Investigating the impact of ComBat harmonization on the reproducibility of MRI radiomic features for different scanners and acquisition parameters with different image pre-processing techniques using a dedicated MRI phantom.

## Materials and methods

**Fig. 1:**
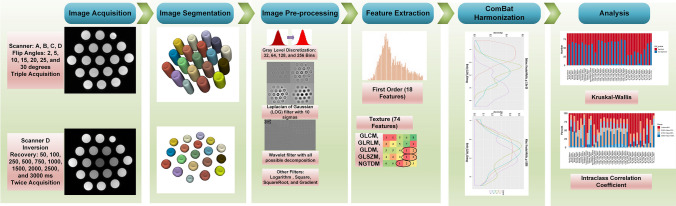
Workflow summarizing the different steps involved in the current study

Different steps involved in the implementation of the current study are shown in Fig. 1.

### Phantom design

The nonanatomic MRI phantom from TCIA (RIDER database), containing 18 gel-filled tubes (25 mm) and one 20-mm tube filled with 0.25 mM GdDTPA, was used in this study [26–28].

### Evaluated scanners

MR images of the above phantom were acquired on 4 scanners by RIDER database. The description of scanners and protocols are summarized in Table 1. Scanner A was chosen for multiple FAs and three times tests, whereas scanner D was selected for multiple IRs to assess the impact of various FAs, tests, and IRs in one scanner owing to the availability of a large number of images on this scanner.

**Table 1:** Description of MRI scanners and protocols used in the current study

Name	Scanner	MRI coils	Gradient specifications	Field-of-view
Scanner A	GE 1.5T	8 Channel HD	BRM gradient subsystem (33 mT/m amplitude; 120 T/m-s)	24 × 19 cm
Scanner B	GE 1.5T	8 Channel HD	CRM gradient subsystem (50 mT/m amplitude; 150 T/m-s)	24 × 19 cm
Scanner C	Siemens 1.5T	8 Channel HD	Espree (VB13) 33 mT/m amplitude, 100 T/m-s gradient subsystem	24 × 19 cm
Scanner D	GE 3.0T	8 Channel HD	TwinSpeed gradients (40 mT/m; 150 T/m-s in zoom mode)	24 × 19 cm

Table 2 shows variables and constant acquisition parameters for 4 analytical approaches 2 [26, 27]. The first analysis was performed on multiple scanners. Here, 4 scanners were used. For each scanner, 3 images with 1-h and 1-week (scanner D 2-week) intervals were acquired. Other constant acquisition parameters are presented in Table 2. Multiple tests were performed on scanner A with triplet tests (1-h and 1-week intervals). Constant acquisition parameters are shown in Table 2. An investigation of multiple FAs with 2, 5, 10, 15, 20, 25, and 30 degrees was performed by a 3D fast spoiled gradient recalled echo (FSPGR) sequence. For each FA, 3 images with 1-h and 1-week intervals were acquired. Other constant values for acquisition parameters are presented in Table 2. Different inversion times in fast spin-echo inversion recovery sequences included 50, 100, 250, 500, 750, 1000, 1500, 2000, 2500, and 3000 ms. For each IR, 2 images with 2-week intervals were acquired. Constant acquisition parameters are shown in Table 2 [26, 27].

**Table 2:** List of variables and constant acquisition parameters for 4 different methods

Evaluation	Variable	Constant
Scanners	Scanner A, Scanner B, Scanner C, Scanner D	T1 Measurements: 3D fast spoiled gradient recalled echo (FSPGR) FA: 20 TE: 1.22 ms TR: 6.38 ms Matrix size: 512 × 512 Slice number: 10 Thickness: 5-mm sections Acquisition time per FA: 0:58 sec
Multiple test	Triplet with 1 hour and 1-week interval	Scanner A T1 Measurements: 3D FSPGR FA: 20 TE: 1.22 ms TR: 6.38 ms Matrix size: 512 × 512 Slice number: 10 Thickness: 5-mm sections Acquisition time per FA: 0:58 sec
Flip angles	2, 5, 10, 15, 20, 25, and 30 degrees	Scanner A T1 Measurements: 3D FSPGR TE: 1.22 ms TR: 6.38 ms Matrix size: 512 × 512 Slice number: 10 Thickness: 5-mm sections Acquisition time per FA: 0:58 sec
Inversion recovery	50, 100, 250, 500, 750, 1000, 1500, 2000, 2500, and 3000 ms.	T1 measurements: fast spin-echo inversion recovery sequence TE: 8.7 ms TR: 5000 ms Matrix size: 256 × 256 Slice number: 1 Thick: 10-mm sections Acquisition time per inversion time: 4 min and 25 sec

### Image segmentation

Manual segmentation of 19 phantom compartments was performed using 3D Slicer version 4.11 [29]. The FAs series contained 12 slices, where the first and last slice was excluded during manual segmentation owing to the change in intensity in this section. IR images were acquired in a single slice, and each slice was separated and segmented.

### Image pre-processing

Before feature extraction, each image was pre-processed using three methods: (i) Bin discretization (32, 64, 128, and 256 bins), (ii) Laplacian of Gaussian (LOG) filter with 10 sigma’s (0.5 to 5 mm in 0.5-mm increment), (iii) wavelet filter with a combination of low- and high-pass filters in 3-dimensions, and (iv) other filters, including logarithm, square, square root, and gradient. It was not possible to use the LOG filter on IR image series since these series consisted of a single slice. Sixty-four bin discretization has been adopted for features extracted from LOG and wavelet-filtered images. These methods were implemented using PyRadiomics [30], which is compliant with image biomarker standardization initiative (IBSI) guidelines for radiomic analysis [31, 32]. In this study, we used fixed bin numbers for Bin discretization based on our previous study [8]. LOG filter was used for edge detection and extraction of key points on the image [30]. Low sigma refers to a fine filter, whereas higher sigma makes the filter coarser [30]. For the wavelet filter, we used Coiflets 1 from PyWavelet library [33] with 8 decompositions, including LLL, LLH, LHL, LHH, HLL, HLH, HHL, and HHH [30]. Logarithm, square, and square root filters were applied to the image and logarithm, square, and square root were calculated from image intensities [30]. Gradient calculated the gradient magnitude of an image. Further details about the use of filters can be found in [30–32].

### Radiomic features extraction

Ninety-two features were extracted within each ROI of phantom images using the IBSI-compatible [34] PyRadiomics package [30] in Python for each pre-processing method, including two feature sets: first-order (FO, 18 features) and textures which also included (i) gray level co-occurrence matrix (GLCM, 23 features), (ii) gray level run length matrix (GLRLM, 16 features), (iii) gray level dependence matrix (GLDM, 14 features), (iv) gray level size zone matrix (GLSZM, 16 features), and (v) neighboring gray tone difference matrix (NGTDM, 5 features).

### ComBat harmonization

The ComBat harmonization method, which Johnson et al. first proposed, assumes that the feature value of y calculated in VOI j and batch (scanner, test, FA, or IR) i is calculated using Eq. (1) [20]:

1 $${y}_{ij}=\alpha +X{}_{ij}{}\beta +{\gamma}_i+{\delta}_i{\varepsilon}_{ij}$$

Accordingly, X indicates a design vector (matrix) for biological covariate(s) of interest, whereas α and β stipulate standard linear regression coefficients [20]. In addition, γi captures the additive batch effect on features (normal distribution assumption), while *δ*_*i*_ captures the multiplicative batch effect (inverse gamma distribution assumption) and *ε*_*ij*_ represents an error part (assumed to have zero-mean normal distribution) [20].

The method below was developed by Fortin et al. [21, 22] using an empirical Bayes model which estimates *γ*_*i*_ and *δ*_*i*_ parameters (denoted as *γ*_i_* and *δ*_i_*), with the normalized feature value of y for VOIj and batch i as follows [20–22]:

2 $${y}_{ij}^{Combat}=\frac{y_{ij}-\hat{\alpha}-X{}_{ij}{}\hat{\beta}-{\gamma_i}^{\ast }}{{\delta_i}^{\ast }}+\hat{\alpha}+X{}_{ij}{}\hat{\beta}$$

wherein *α* and *β* parameters were estimated and noted as $\hat{\alpha}$ and $\hat{\beta}$ in Eq. (2), respectively [21, 22]. It is worth mentioning that ComBat harmonization employs a transformation method for each feature, which is separately governed by the batch effect observed on feature values [21, 22]. As such, a non-parametric model with an empirical Bayes estimation of the ComBat method was applied, revealing no biological covariates and no assumptions for *γ*_*i*_, *δ*_*i*_, and *ε*_*ij*_. The ComBat R function^1^ used in this study is publicly available [21, 22].

### Data analysis

The investigation of the ComBat harmonization effect on feature values was performed by Kruskal-Wallis’s one-way test. This test was applied to features before and after harmonization among multiple batches. The batches here defined multiple scanners, tests, FAs, and IRs. A *p*-value less than 0.05 was considered statistically significant. Features with a significant *p*-value indicate a significant difference between batches, whereas a non-significant *p*-value indicates no significant difference between batches. The intraclass correlation coefficient (ICC) calculation was performed for each individual radiomic feature over a varoius batch with a random two-way effects model. ICC values were categorized into 4 groups, (i) 90% ≤ ICC ≤ 100%, (ii) 75% ≤ ICC < 90%, (iii) 50% ≤ ICC < 75%, and (iv) ICC < 50% [35]. Radiomic features showing ICC > 90% were selected as robust features against each effective factor. We used irr package version 0.84.1 for ICC and stats package for the KW test in R version 4.0.4 (The R Foundation, Vienna, Austria) [36].

## Results

**Fig. 2:**
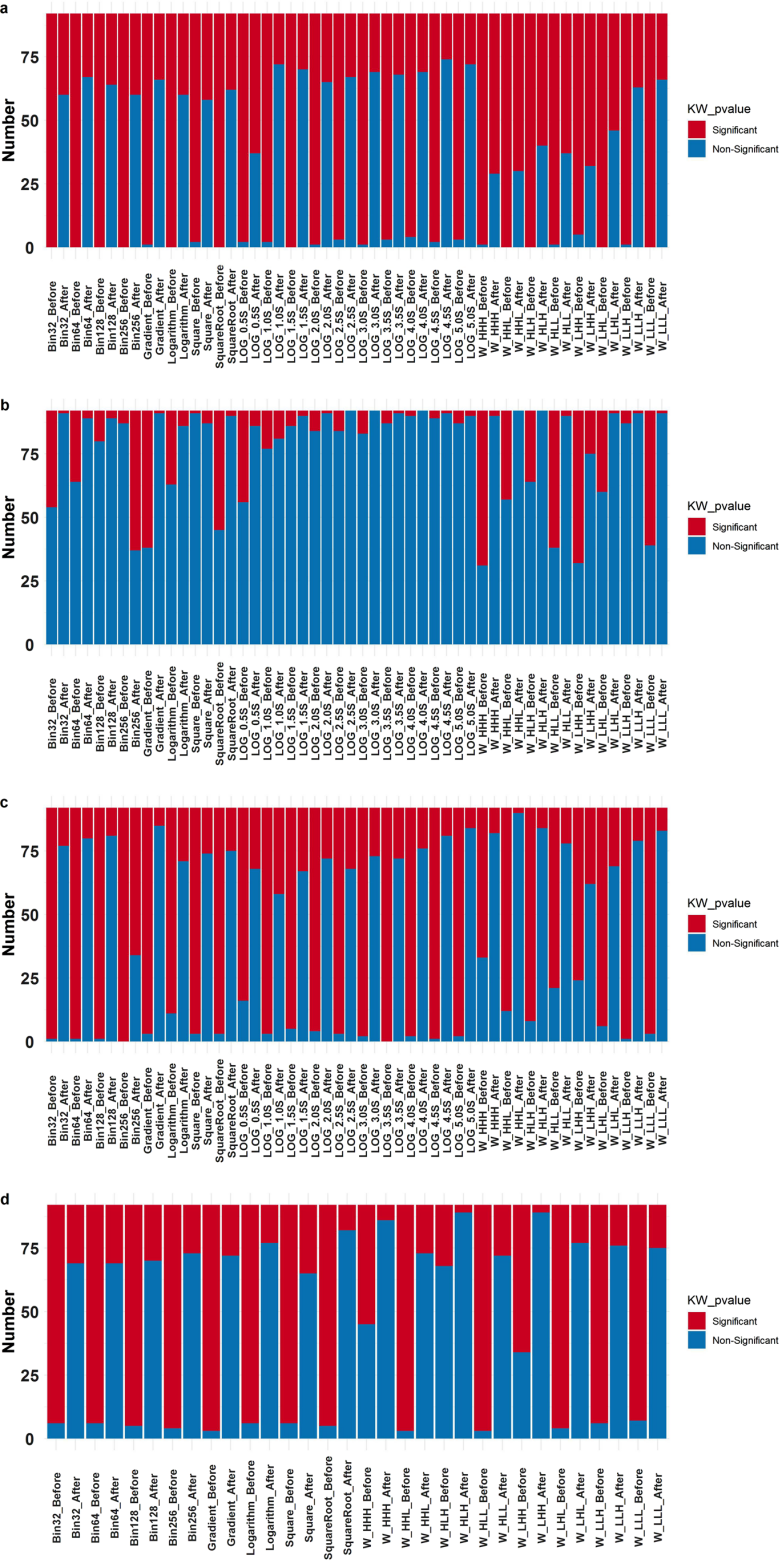
Results of Kruskal-Wallis’s (KW) test before and after ComBat harmonization, over various image pre-processing techniques for MR images acquired on: **a** four scanners, **b** three times test, **c** various flip angles, and **d** various inversion recoveries. LOG, Laplacian of Gaussian (LOG); S, sigma; W, wavelet; L, low-pass filter; H, high-pass filter

Figure 2a depicts the KW test among four scanners before and after ComBat harmonization when using various image pre-processing techniques. The number of features with non-significant *p*-values (lower variability) ranged between 0–5 and 29–74 before and after ComBat harmonization along with various image pre-processing techniques, respectively. The LOG filter with 4.5-mm sigma had the highest non-significant *p*-values (2 before, 74 after). Other LOG filters performed better than other methods (65–72 features after).

Figure 2b shows the KW test among three times tests before and after ComBat harmonization using various image pre-processing steps. The number of non-significant features set is considerably higher than the results of the KW test evaluating other factors, where W_HHH_Before, W_LHH_Before, and Bin256_After had the highest number of significant *p*-value features with 61, 60, and 55 features, respectively. Besides, LOG_2.5S, LOG_3.0S, LOG_4.0S, W_HHL, and W_HLH, after ComBat harmonization, had non-significant *p*-value features, thus demonstrating the constructive impact of ComBat harmonization on features variability. These feature sets had 84, 83, 90, 57, and 64 non-significant features before ComBat harmonization.

Figure 2c shows the KW test among multiple FA before and after ComBat harmonization when using various image pre-processing techniques. The number of features with non-significant *p*-values ranged from 0–33 to 34–90 before and after ComBat harmonization along with different image pre-processing steps, respectively. The wavelet filter with HHL decomposition had the highest number of non-significant *p*-values (12 before and 90 after), followed by the gradient filter, LOG filter with 5.0-mm sigma, and wavelet filter with HLH decomposition, which had 85, 84, and 84 non-significant features after ComBat harmonization. Other wavelet filters performed better than other methods (62–84 features after).

Figure 2d depicts the KW test among multiple IR before and after ComBat harmonization when using various image pre-processing techniques. The number of features with non-significant *p*-values ranged from 3–68 to 65–89 before and after ComBat harmonization along with other image pre-processing techniques, respectively. The wavelet filter with HLH, LHH, and HHH decompositions had the highest non-significant *p*-values before (68, 34, and 45 features) and after (89, 89, and 86 features) ComBat harmonization using various image pre-processing techniques, respectively. The square root filter had 82 non-significant features after ComBat harmonization.

Supplemental Figures 1-4 depict the KW test results for each radiomic feature before and after ComBat harmonization using various image pre-processing techniques. Supplemental Table 1 shows which radiomic feature had over 20 non-significant (over 15 for IR) in scanners, three times repeated the test, various flip angles, and inversion recovery across various image pre-processing techniques after ComBat harmonization. Supplemental Figures 5-8 indicate that instance radiomic features were common in 4 analyses. Table 3 shows the numbers of non-significant features before and after ComBat harmoniztion, along with various image pre-processing techniques.

**Table 3:** Comparison of the number of non-significant features before and after ComBat harmonization when using different image pre-processing techniques

Feature set	Scanner		Three times test		FA		IR	
	Before	After	Before	After	Before	After	Before	After
Bin32	0	60	54	91	1	77	6	69
Bin64	0	67	64	89	1	80	6	69
Bin128	0	64	80	89	1	81	5	70
Bin256	0	60	87	37	0	34	4	73
Gradient	1	66	38	91	3	85	3	72
Logarithm	0	60	63	86	11	71	6	77
Square	2	58	91	87	3	74	6	65
SquareRoot	0	62	45	90	3	75	5	82
LOG_0.5S	2	37	56	86	16	68		
LOG_1.0S	2	72	77	81	3	58		
LOG_1.5S	0	70	86	90	5	67		
LOG_2.0S	1	65	84	91	4	72		
LOG_2.5S	3	67	84	92	3	68		
LOG_3.0S	1	69	83	92	2	73		
LOG_3.5S	3	68	87	91	0	72		
LOG_4.0S	4	69	90	92	2	76		
LOG_4.5S	2	74	89	91	1	81		
LOG_5.0S	3	72	87	90	2	84		
W_HHH	1	29	31	90	33	82	45	86
W_HHL	0	30	57	92	12	90	3	73
W_HLH	0	40	64	92	8	84	68	89
W_HLL	1	37	38	90	21	78	3	72
W_LHH	5	32	32	75	24	62	34	89
W_LHL	0	46	60	91	6	69	4	77
W_LLH	1	63	87	91	1	79	6	76
W_LLL	0	66	39	91	3	83	7	75

*FA filp angle, *IR* inverstion recovery, *LOG* Laplacian of Gaussian (LOG), *S* sigma, *W* wavelet, L low-pass filter, *H* high-pass filter

**Fig. 3:**
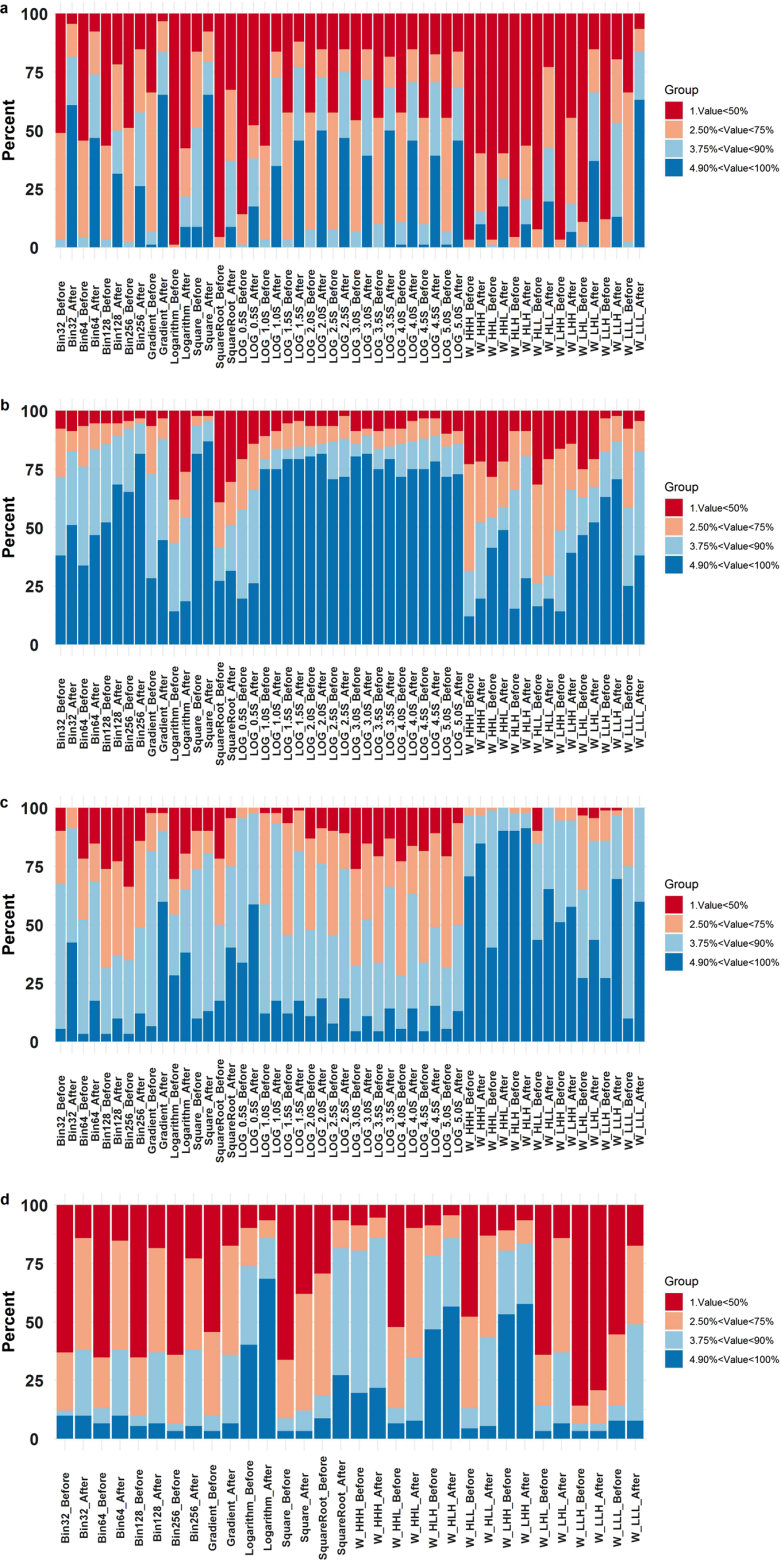
ICC percentage value of different radiomic features set over: **a** various scanners, **b** three times repeated tests, **c** various flip angles, and **d** inversion recovery before and after ComBat harmonization

In Fig. 3, we illustrate the ICC percentage of various radiomic feature sets before and after ComBat harmonization. We also show the number of robust features with ICC ≥ 90% in different feature sets before and after harmonization in Table 4. It is evident from Fig. 3a that the impact of different scanners on the LOG feature set and different wavelet feature sets are the least and the most, respectively. Furthermore, ComBat harmonization affects the reproducibility of the radiomic feature set against all parameters, where 80% of feature sets had no robust features (ICC ≥ 90%) before ComBat harmonization. Furthermore, Logarithm_Before, SquareRoot_Before, W_HHH_Before, W_HLH_Before, W_HLL_Before, W_LHH_Before, and W_LLH_Before features sets had no features in the third group (90% < ICC ≥ 75%) either. All of the feature sets showed robust features after ComBat harmonization. After ComBat harmonization, W_LHH_After showed the least reproducibility with 6 robust features, whereas Square_After and Gradient_After led to the highest number of robust features with 60 features. As observed in Fig. 3b, three times, the test showed the most negligible impact on the radiomic features set, and Square feature sets showed the most robustness with 75 and 80 features before and after ComBat harmonization. Different wavelet feature sets are the most robust features against various FA (Fig. 3c). In Fig. 3d, the Logarithm_After features set with 63 robust features showed the most reproducibility, and W-LLH_After showed the least reproducibility with 3 robust features over various IR after ComBat harmonization.

**Table 4:** Comparison of the number of rubust features (ICC > 90%) before and after ComBat harmonization when using different image pre-processing techniques

Feature set	Scanner		Three times test		FA		IR	
Set	Before	After	Before	After	Before	After	Before	After
Bin32	0	56	35	47	5	39	9	9
Bin64	0	43	31	43	3	16	6	9
Bin128	0	29	48	63	3	9	5	6
Bin256	0	24	60	75	3	11	3	5
Gradient	1	60	26	41	6	55	3	6
Logarithm	0	8	13	17	26	35	37	63
Square	8	60	75	80	9	12	3	3
Square root	0	8	25	29	16	37	8	25
LOG_0.5S	0	16	18	24	31	54		
LOG_1.0S	0	32	69	69	11	16		
LOG_1.5S	0	42	73	73	11	16		
LOG_2.0S	0	46	74	75	10	17		
LOG_2.5S	0	43	65	66	7	17		
LOG_3.0S	0	36	74	75	4	10		
LOG_3.5S	0	46	69	73	4	13		
LOG_4.0S	1	42	66	69	5	13		
LOG_4.5S	1	36	69	72	4	14		
LOG_5.0S	1	42	66	67	5	12		
W_HHH	0	9	11	18	65	78	18	20
W_HHL	0	16	38	45	37	83	6	7
W_HLH	0	9	14	26	83	84	43	52
W_HLL	0	18	15	18	40	60	4	5
W_LHH	0	6	13	36	47	53	49	53
W_LHL	0	34	43	48	25	40	3	6
W_LLH	0	12	58	65	25	64	3	3
W_LLL	0	58	23	35	9	55	7	7

*FA filp angle, *IR* inverstion recovery, *LOG* Laplacian of Gaussian (LOG), *S* sigma, *W* wavelet, *L* low-pass filter, *H* high-pass filter

Supplemental Figures 9-12 show the ICC heat map of radiomic features with different image pre-processing techniques over various scanners, three times repeated tests, various flip angles, and inversion recovery before and after ComBat harmonization, respectively.

**Fig. 4:**
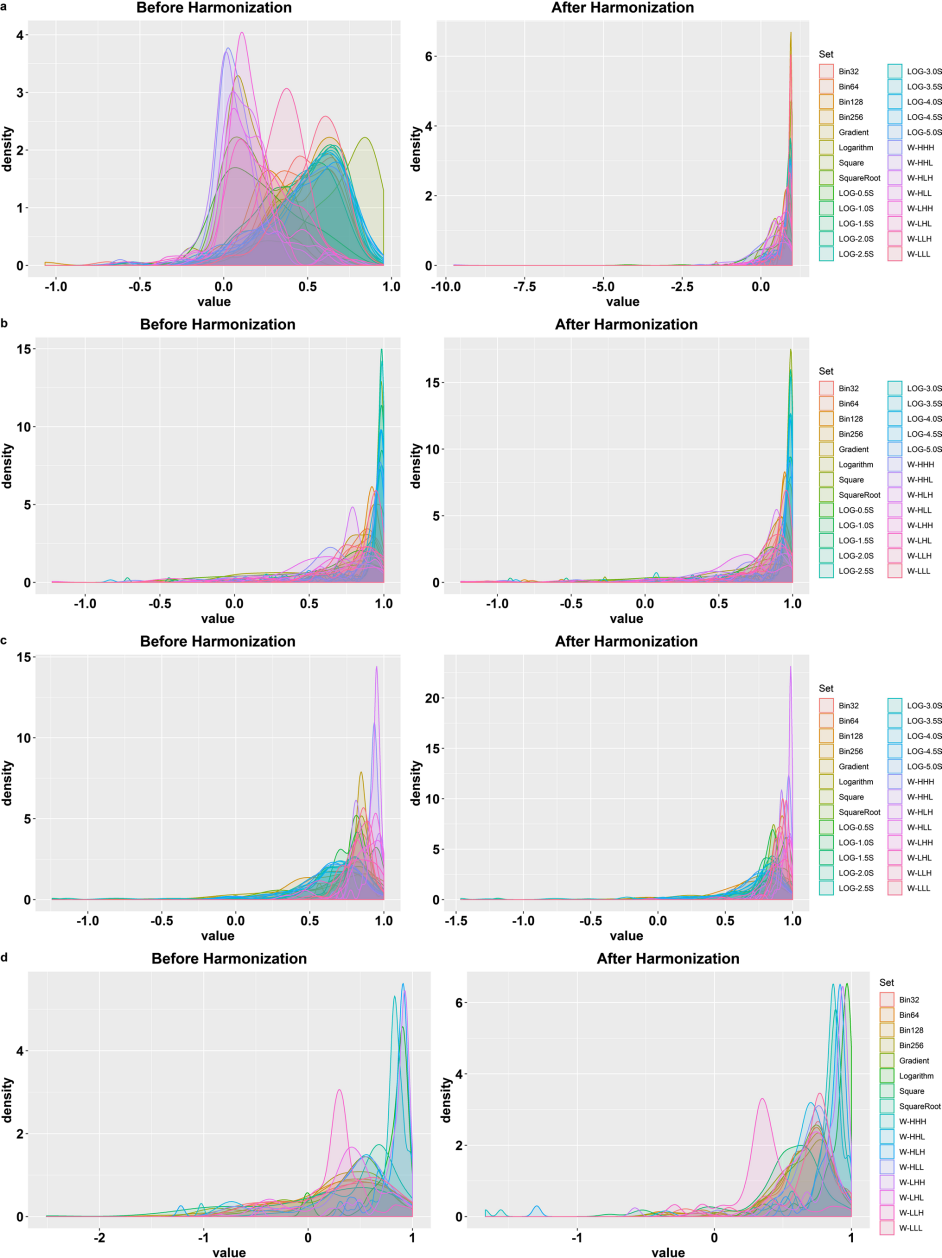
ICC value density plots (DPs) of the different radiomic feature sets over various scanners (**a**), three LOG: Laplacian of Gaussian (LOG), S, sigma; W, wavelet; L, low-pass filter; H, high-pass filter, times repeated test (**b**), flip angles (**c**), and inversion recovery (**d**), before (left panel) and after (right panel) ComBat harmonization. LOG, Laplacian of Gaussian (LOG); S, sigma; W, wavelet; L, low-pass filter; H, high-pass filter

Figure 4a depicts the impact of multiple scanners on radiomic features where the distribution of ICC values is scaled between − 1 and + 1. When the density graph is left uneven, the mean is less than the median in the density plot (DP). Conversely, the plot concentration on the right panel (+ 1) illustrates the robustness. The left panel in Fig. 4a shows the massive distribution of ICC values representing the low reproducibility of radiomic features set over the different scanners before ComBat harmonization. The right panel of the same figure (after ComBat harmonization) indicates the beneficial effect of ComBat harmonization on the radiomic features set robustness. Figure 4b shows that most of the feature sets had high reproducibility before and after harmonization. Figure 4c and d illustrate the ICC values concentration before and after ComBat harmonization over various FAs and IR. Although the impact of FA/IR is less than when using multiple scanners, the DP of radiomic features turns left after ComBat harmonization, which proves the constructive effect of ComBat harmonization on the reproducibility of the radiomic features set.

## Discussion

We investigated the effect of ComBat harmonization on radiomic features extracted from MRI phantom when applying different image pre-processing techniques on images acquired using various imaging protocols (multiple FAs and IRs) and scanners. The results indicated that ComBat harmonization decreased the variability of radiomic features across various multi-center images and imaging protocols acquired on different scanners. In addition, using different image pre-processing techniques reduced the radiomic features’ variability.

The results of the KW test indicate that the number of non-significant radiomic feature sets will rise after applying ComBat harmonization regardless of image pre-processing techniques applied in the current study. Following ComBat harmonization, LOG_4.5S, LOG_1.0S, and LOG_5.0S had the highest number of non-significant radiomic feature sets over multiple scanners in the KW test (Fig. 2) with 74, 72, and 72 non-significant features, respectively. Besides, prior to ComBat harmonization, Bin32, Bin64, Bin128, Bin256, logarithm, square root, LOG_1.5S, W_HHL, W_HLH, W_LHL, and W_LLL had no significant radiomic features. MRI scanners have the largest impact on radiomic features among the parameters investigated in the current study. The use of various MRI scanners showed the largest effect on radiomic features before and after ComBat, even more than IR and FA combined. Ninety-four percent of radiomic features that are robust against FA and IR simultaneously are also robust against different scanners. Three times test had the most negligible impact on radiomic features variability.

Concerning the effects of image pre-processing on the variability of radiomic features, the study by Demircioğlu et al. [37] used public radiomic datasets to investigate the effect of various pre-processing filters on the predictive performance of radiomic models. They found that adding features pre-processed with various filters improved the predictive performance, although using pre-processing filters in some datasets showed the opposite [37]. Tuning the filters further improved the results, indicating that pre-processing filters should be used in radiomic studies to improve the predictive performance [37]. Moradmand et al. [38] investigated the impact of pre-processing techniques on MRI radiomic features and reported that 23% of radiomic features after bias field correction were robust (ICC > 90%). Yet, overlooking inter-scanner, inter-vendor, and inter-protocol variations in radiomics research can not only adversely affect the results but may also lead to failure in the process of finding uncertainties in radiomics research. In spite of the significance of such deviations, only few studies have investigated this sphere and identified precautionary measures.

In a study conducted by Orlhac et al. [1], the RIDER MRI phantom scanned on 1.5 T and 3 T scanners were used to extract 42 radiomic features, 40 of which had significant differences prior to ComBat harmonization. Following ComBat harmonization, this number was reduced to 0 features. The same phantom data was used in the present work, but contrary to the above reference, we used all available scanners, including three 1.5 T scanners and one 3 T scanner, besides extracting all 3D IBSI radiomic features and implementing various image pre-processing techniques, including different discretization of bins (32, 64, 128, and 256 bins), logarithm, square, square root, and gradient filters, LOG filter with 10 sigmas, and wavelet filter with 8 decompositions. Furthermore, all data used in the current study were acquired three times using multiple flip angles and inversion recovery settings. Our findings confirm the results of this study, i.e., all features with different bin discretization (32, 64, 128, and 256) had significant differences before scanner harmonization. However, this number varied in other pre-processing methods (0–5 features had non-significant differences).

Furthermore, our results demonstrated that the best feature set was the LOG filter (4.5-mm sigma), with 74 features with non-significant differences in different scanners. In a recent study, Li et al. [3] investigated how pre-processing steps and harmonization procedures (such as the ComBat method for radiomic features) may reduce scanner effects and enhance radiomic features’ repeatability in brain MRI radiomics. Their findings are entirely in line with ours in the sense that ComBat harmonization might increase radiomic features reproducibility to a large extent over various image pre-processing steps.

Another noteworthy finding that is highlighted in our results is that several times testing turned out to have the most negligible impact on radiomic features. In the context of radiomics, imaging at multiple time points enables researchers to analyze features’ robustness to temporal variabilities, e.g., organ expansion, shrinkage, and motion [39]. However, the significance of temporal variations fades in relation to inter-scanner variations, which are capable of causing much more fundamental inconsistencies between samples [40]. Lee et al. [41] investigated the robustness of radiomic features in an MRI phantom. The ICC for test-retest analysis in phantoms with different materials was reported to be high (average ICC = 0.96 for T1-w images). While our study employed a three-time test, the ICC was also high for the majority of pre-processing methods, such as the LOG filter with different sigma.

Few previous studies explored the impact of image pre-processing techniques and ComBat harmonization at the same time and managed to follow concise protocols [3, 10, 42]. Nevertheless, there is a key point to keep in mind when interpreting the present findings since Baeßler et al. [4] showed in a phantom study that the number of robust features in a FLAIR MR image is higher than in T1- and T2-weighted images. Consequently, it is expected that different MR sequences might affect radiomic features, which was not explored in the current study.

Alternative methods for feature extraction, such as deep learning-based feature [43], Bag of Features (BoF) [44] and Local Binary Patterns (LBPs) [45] were also reported in the literature. These techniques were not used in the current study. Further analysis is required to explore the effect of various feature extraction methods. Our findings underscore the importance of harmonization in MRI radiomics, potentially enhancing diagnostic accuracy and reliability in multi-center studies. By reducing variability across different scanners and protocols, ComBat harmonization could lead to more consistent radiomic features, improving patient care through better-informed diagnostic and prognostic models. This advancement holds promise for standardized imaging biomarkers in clinical practice, offering a path toward more personalized and precise medical interventions.

## Conclusion

ComBat harmonization appears to be a decent solution to enhance MRI radiomic features reproducibility. The use of multiple scanners had the highest impact on radiomic features variability, followed by IR and FA. Most of the robust features against scanners are robust against IR and FA. However, acquiring several test images on a single scanner had the lowest impact on radiomic features among the remaining parameters. The main contribution of the current study is the consideration of various image pre-processing and data acquisition protocols using different scanners and 3 times repeated scanning to avoid any errors. However, our study inherently bears some limitations, the main one being that the effect of different MR imaging protocols was overlooked. Future studies will evaluate the effect of other MRI scanning protocols on the reproducibility of radiomic features to tackle this limitation. Another limitation is the use of only phantom images. Clinical studies are required to validate these results.

## Supplementary information

ESM 1 (PDF 3878 kb)

## Data Availability

The data used in this study are publically available from the TCIA archive. (10.7937/K9/TCIA.2015.MI4QDDHU).

## Footnotes

1 https://github.com/Jfortin1/ComBatHarmonization

## Code availability

Open-source libraries used in this study. PyRadiomics (https://github.com/AIM-Harvard/pyradiomics) for image pre-processing and feature extraction in Python, ComBat harmonization (https://github.com/Jfortin1/ComBatHarmonization) in R and irr and stats packages for statistical analysis.
