# Supplementary material for: Impact of harmonization on the reproducibility of MRI radiomic features when using different scanners, acquisition parameters, and image pre-processing techniques: a phantom study
Source: Med Biol Eng Comput. 2024 Mar 27;62(8):2319–32. doi: 10.1007/s11517-024-03071-6 (PMC11604802; doi:10.1007/s11517-024-03071-6)
Supplement: Supplementary file 1 — (PDF 3878 kb) [file 11517_2024_3071_MOESM1_ESM.pdf]

Table 1. Radiomic features had over 20 non-significant (over 15 for IR) in different scanners, three times repeated tests, various flip angles (FA), and inversion recovery (IR) across different image pre-processing techniques after ComBat harmonization.

Scanner		Test		FA		IR	
**Feature**	**No.**	**Feature**	**No.**	**Feature**	**No.**	**Feature**	**No.**
FO_MAD	25	FO_10Percentile	26	FO_Energy	26	FO_90Percentile	16
FO_Range	25	FO_90Percentile	26	FO_MAD	26	FO_Energy	16
FO_RMAD	25	FO_Energy	26	FO_Range	26	FO_Maximum	16
FO_RMS	25	FO_IQR	26	FO_TE	26	FO_MAD	16
FO_Maximum	24	FO_MAD	26	GLDM_LDE	26	FO_RMAD	16
GLSZM_ZE	24	FO_Range	26	GLRLM_RV	26	FO_TE	16
GLCM_Id	23	FO_RMAD	26	FO_RMAD	25	FO_Variance	16
GLCM_IDM	23	FO_RMS	26	FO_RMS	25	GLCM_CT	16
GLCM_JA	23	FO_TE	26	GLCM_DA	25	GLCM_Contrast	16
GLCM_SA	23	FO_Variance	26	GLCM_Id	25	GLCM_DA	16
GLCM_SE	23	GLDM_DE	26	GLCM_IDM	25	GLCM_DE	16
GLCM_JEntropy	22	GLDM_LDE	26	GLDM_DV	25	GLCM_JEntropy	16
FO_Mean	21	GLRLM_RLNUN	26	GLSZM_SALGLE	25	GLRLM_GLV	16
GLCM_DE	21	GLRLM_RP	26	GLSZM_ZP	25	GLRLM_RE	16
GLRLM_RP	21	FO_Minimum	25	GLDM_SDLGLE	24	GLSZM_ZP	16
FO_10Percentile	20	GLCM_Correlation	25	GLSZM_LGLZE	24	FO_Mean	15
GLCM_CT	20	GLCM_Id	25	FO_Skewness	23	GLDM_DNUN	15
GLDM_LDHGLE	22	GLDM_SDE	26	GLRLM_RLNUN	25	GLCM_MP	16
GLCM_DA	20	GLCM_IDM	25	GLCM_SE	23	GLDM_SDHGLE	15
GLRLM_RE	22	GLRLM_LRE	26	GLSZM_GLNUN	25	GLCM_SS	16
FO_IQR	24	FO_Mean	26	GLDM_DE	26	FO_Range	16
GLCM_AC	24	FO_Median	26	GLRLM_RP	26	FO_RMS	16
GLDM_HGLE	23	GLDM_DNU	26	GLCM_IV	25	GLCM_DV	16
GLSZM_GLNU	23	GLDM_DNUN	26	GLDM_DNU	25	GLCM_Id	16
GLSZM_SAE	23	GLDM_DV	26	GLDM_DNUN	25	GLCM_IDM	16
GLRLM_HGLRE	22	GLDM_SDLGLE	26	GLRLM_SRE	25	GLCM_SE	16
GLCM_MP	21	GLRLM_SRLGLE	26	GLCM_Contrast	24	GLSZM_GLV	16
GLSZM_LGLZE	22	GLRLM_RE	26	GLSZM_SAE	25	GLDM_GLV	16
GLSZM_SALGLE	22	GLRLM_RLNU	26	GLSZM_SAHGLE	25	GLDM_SDE	16
GLCM_IV	21	GLRLM_RV	26	FO_IQR	24	GLRLM_RLNU	16
GLCM_JEnergy	21	GLRLM_SRE	26	FO_Kurtosis	24	GLRLM_RP	16
GLDM_DE	21	FO_Maximum	25	GLCM_JEntropy	24	GLSZM_SZNU	16
FO_90Percentile	20	GLCM_DA	25	GLSZM_SZNUN	24	FO_Skewness	15
GLSZM_GLNUN	21	FO_Uniformity	25	GLSZM_GLNU	24	FO_10Percentile	15
FO_Uniformity	20	GLCM_DV	25	FO_Mean	23	GLCM_SA	15
GLSZM_SZNUN	21	GLCM_Contrast	25	GLSZM_LAHGLE	24	FO_Kurtosis	15
FO_Entropy	20	GLCM_DE	25	FO_Entropy	23	GLCM_JA	15

GLCM_IDN	20	GLCM_IDN	25	GLDM_SDE	23	GLRLM_GLNU	15
GLCM_SS	20	GLCM_IMC1	25	GLSZM_ZE	23	GLRLM_LRHGLE	15
GLRLM_GLNUN	20	GLCM_IMC2	25	FO_10Percentile	22	GLRLM_RLNUN	15
GLRLM_SRLGLE	20	GLCM_IV	25	FO_Maximum	22	GLRLM_SRHGLE	15
		GLCM_JEnergy	25	FO_Median	22	GLSZM_HGLZE	15
		GLCM_JEntropy	25	FO_Variance	22	GLSZM_SAHGLE	15
		GLCM_MP	25	GLCM_JEnergy	22	GLSZM_ZE	15
		GLDM_LDLGLE	25	GLRLM_GLNU	22	NGTDM_Strength	15
		GLRLM_GLNU	25	GLRLM_LRE	22		
		GLRLM_LRHGLE	25	GLRLM_RLNU	22		
		GLRLM_LRLGLE	25	GLRLM_SRHGLE	22		
		GLRLM_SRHGLE	25	GLRLM_SRLGLE	22		
		GLSZM_GLNU	25	GLSZM_ZV	22		
		GLSZM_GLNUN	25	GLCM_AC	21		
		GLSZM_GLV	25	GLCM_Correlation	21		
		GLSZM_HGLZE	25	GLCM_DV	21		
		GLSZM_LAE	25	GLCM_IDN	21		
		GLSZM_LAHGLE	25	GLCM_JA	21		
		GLSZM_LALGLE	25	GLCM_SA	21		
		GLSZM_LGLZE	25	GLDM_HGLE	21		
		GLSZM_SAE	25	GLSZM_HGLZE	21		
		GLSZM_SAHGLE	25	GLCM_CT	20		
		GLSZM_SALGLE	25	GLCM_DE	20		
		GLSZM_ZE	25	GLRLM_GLV	20		
		GLSZM_ZP	25	GLRLM_HGLRE	20		
		GLSZM_ZV	25	GLSZM_GLV	20		
		NGTDM_Contrast	25	GLSZM_SZNU	20		
		FO_Entropy	24				
		FO_Kurtosis	24				
		FO_Skewness	24				
		GLCM_CS	24				
		GLCM_CT	24				
		GLCM_IDMN	24				
		GLCM_SE	24				
		GLDM_GLNU	24				
		GLDM_GLV	24				
		GLDM_LDHGLE	24				
		GLDM_LGLE	24				
		GLDM_SDHGLE	24				
		GLRLM_GLNUN	24				
		GLRLM_GLV	24				

		GLRLM_LGLRE	24				
		GLSZM_SZNU	24				
		GLCM_SS	23				
		GLDM_HGLE	23				
		GLRLM_HGLRE	23				
		GLSZM_SZNUN	23				
		NGTDM_Complexity	23				
		GLCM_AC	22				
		GLCM_JA	22				
		GLCM_SA	22				
		NGTDM_Strength	22				

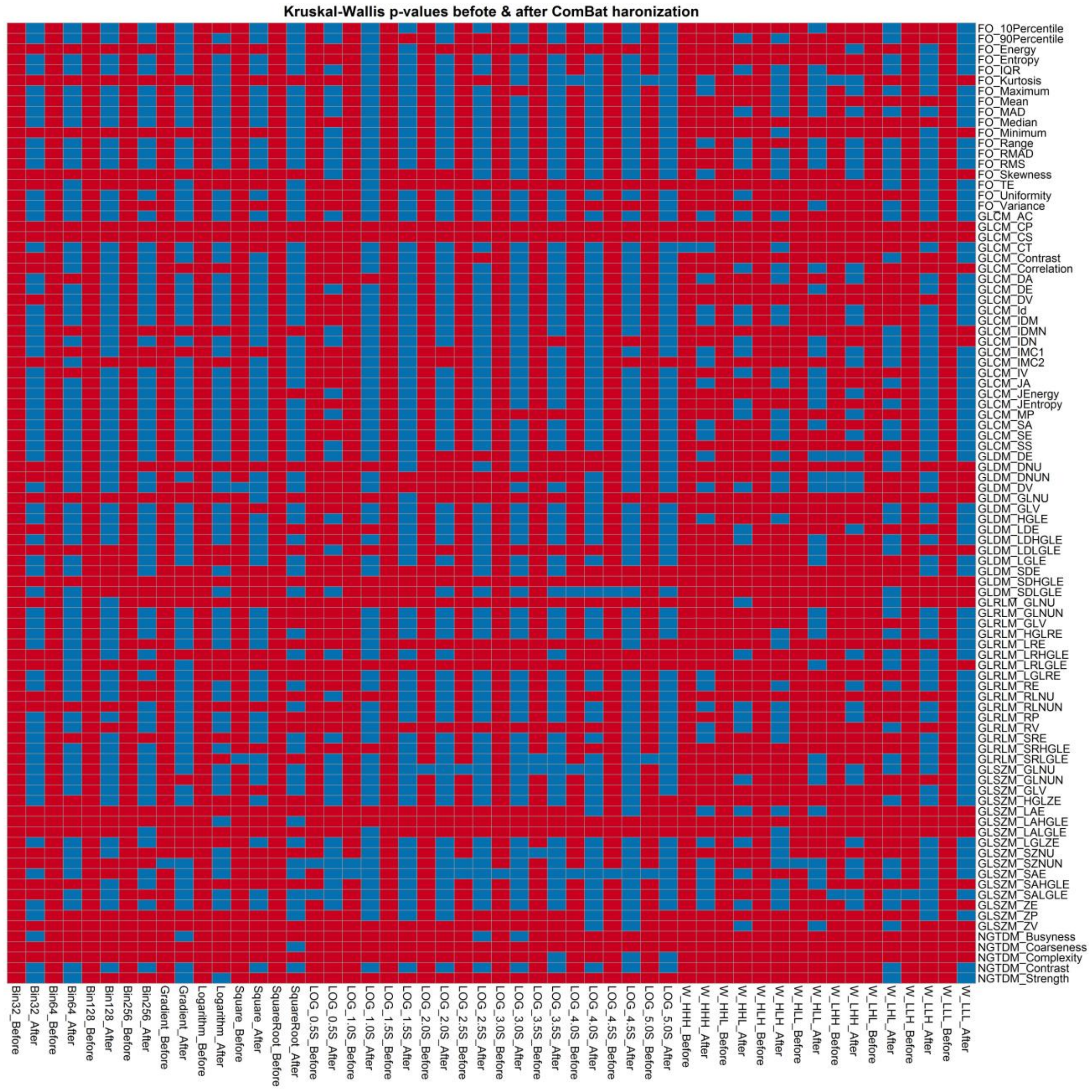

Figure 1. Outcome of the KW test for each radiomic feature before and after ComBat harmonization over various image pre-processing techniques on four scanners.

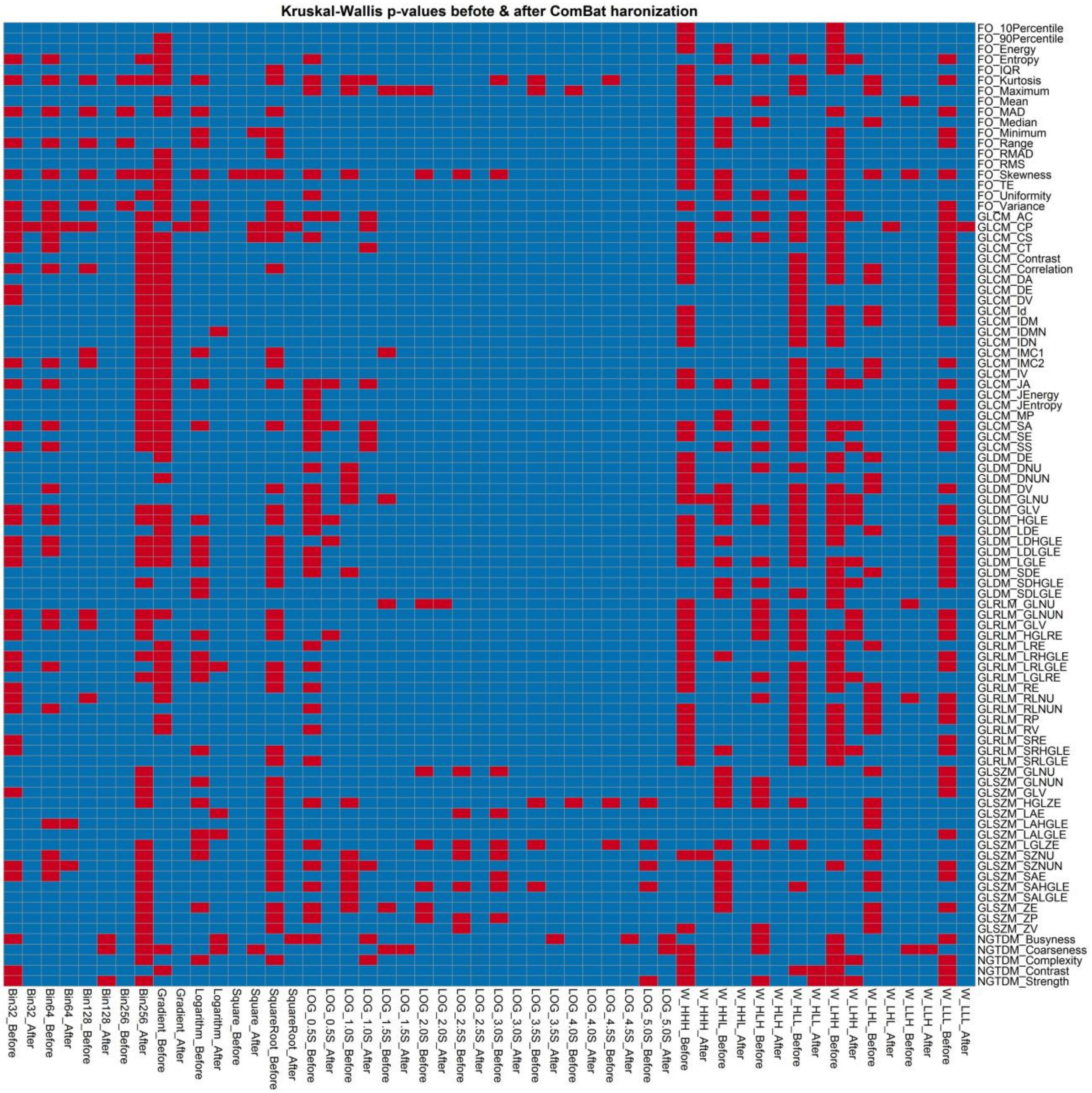

Figure 2. Outcome of the KW test for each radiomic feature before and after ComBat harmonization over various image pre-processing techniques on three times test.

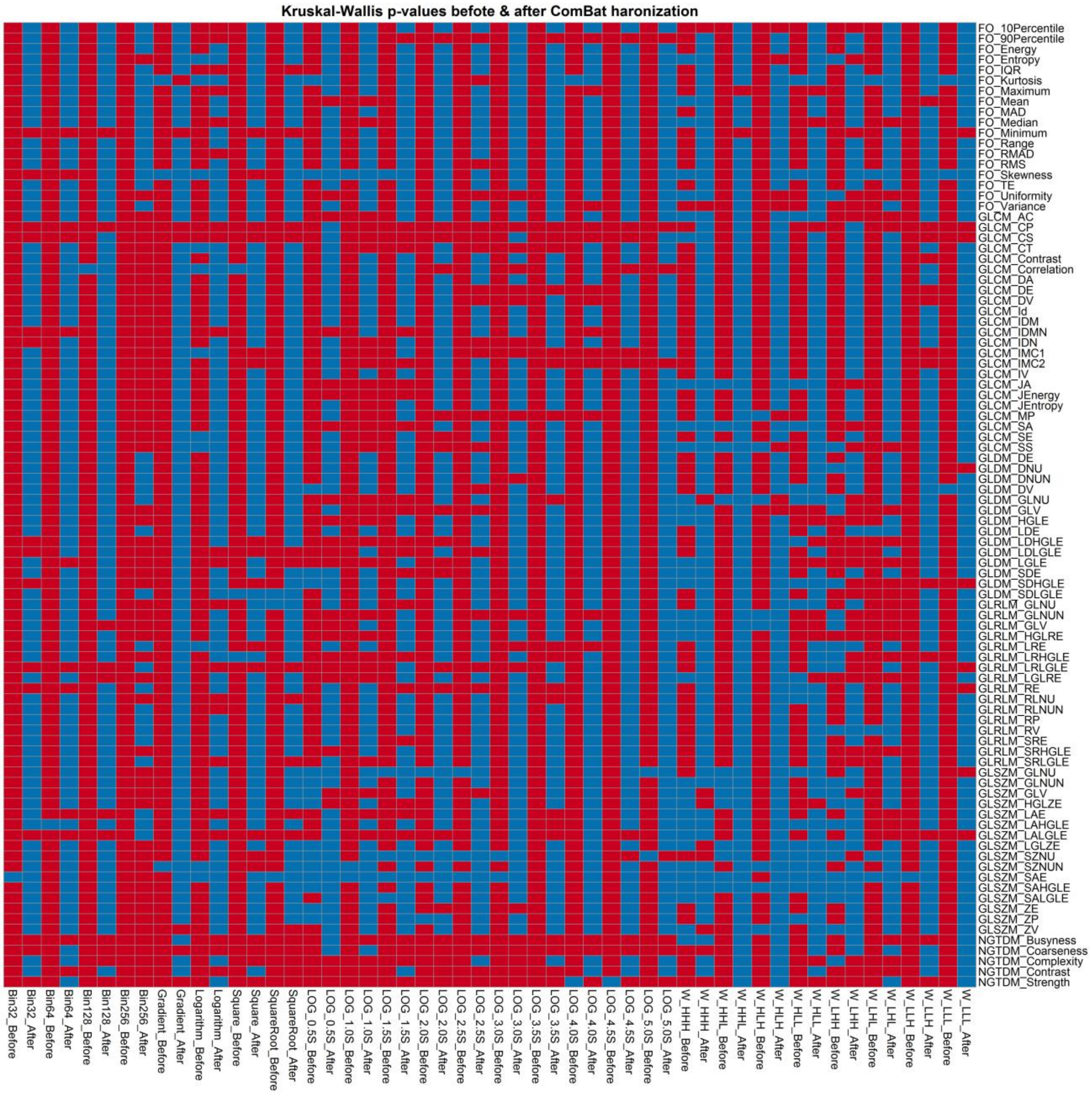

Figure 3. Outcome of the KW test for each radiomic feature before and after ComBat harmonization over various image pre-processing techniques on various flip angles.

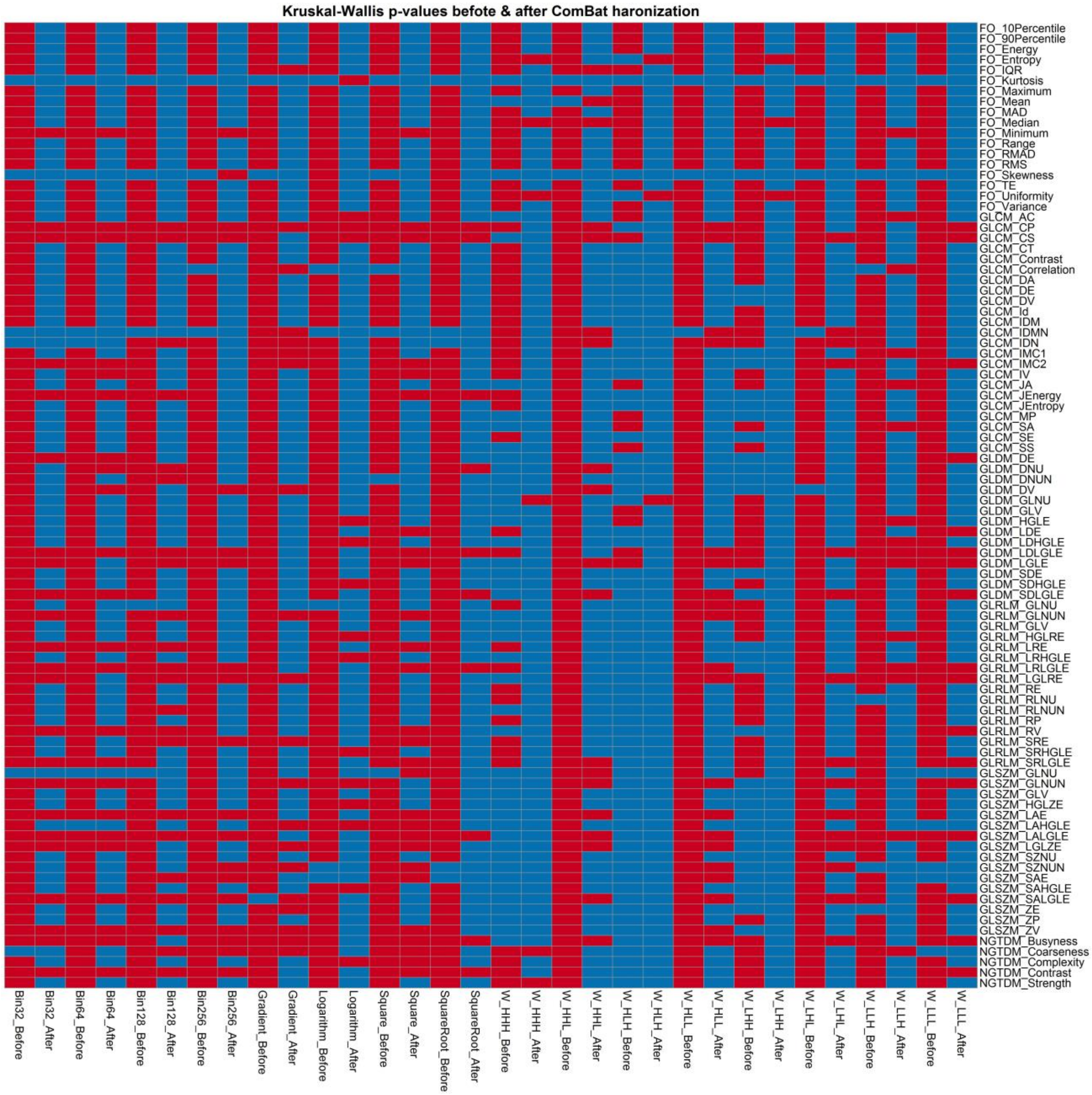

Figure 4. Outcome of the KW test for each radiomic feature before and after ComBat harmonization over various image pre-processing techniques on various inversion recoveries.

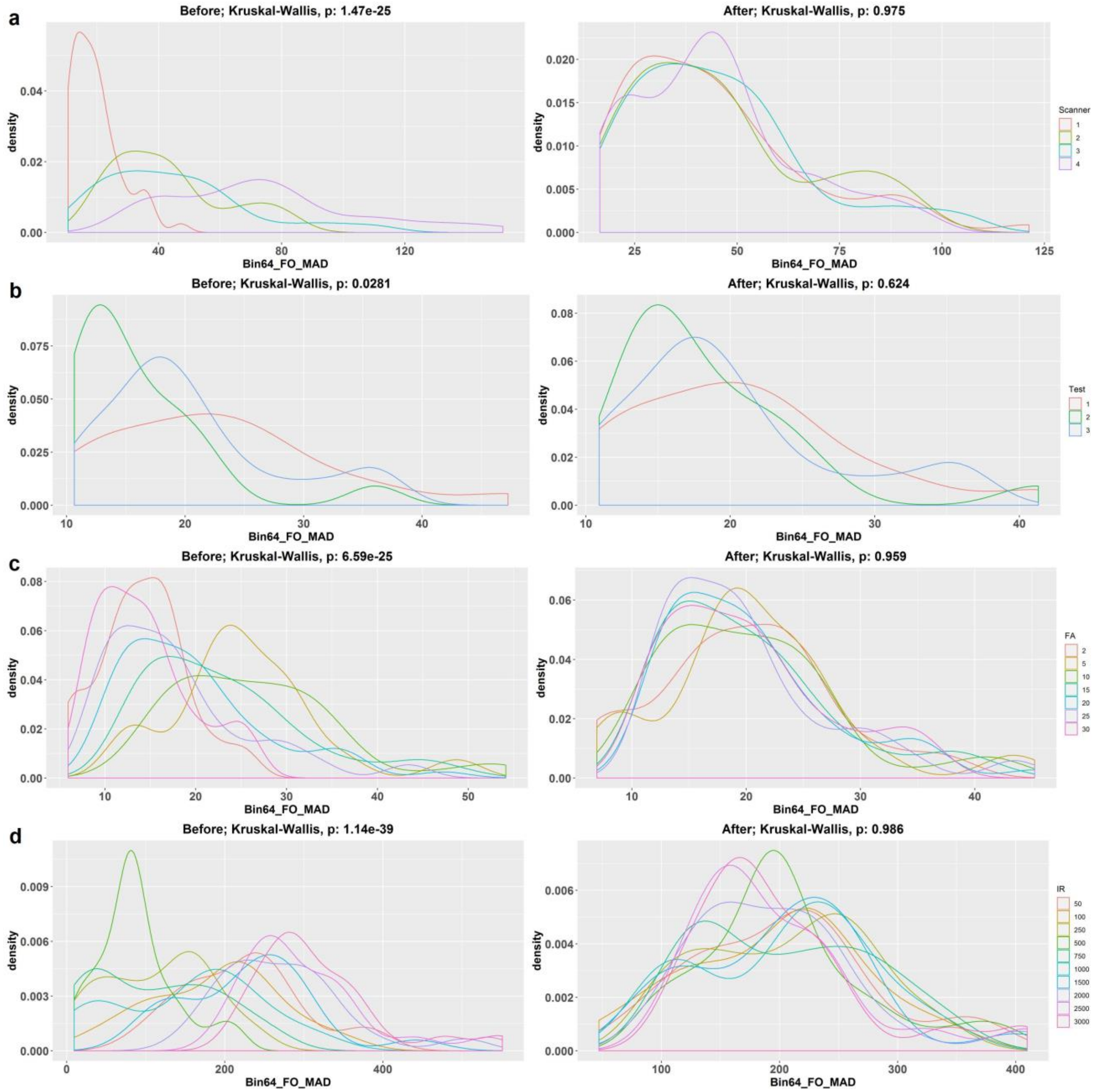

Figure 5. Density plots of Mean Absolute Deviation feature from first-order group before and after ComBat harmonization with 64 bins discretizing on: a) four scanners, b) three times test, c) various flip angles, and d) various inversion recovery.

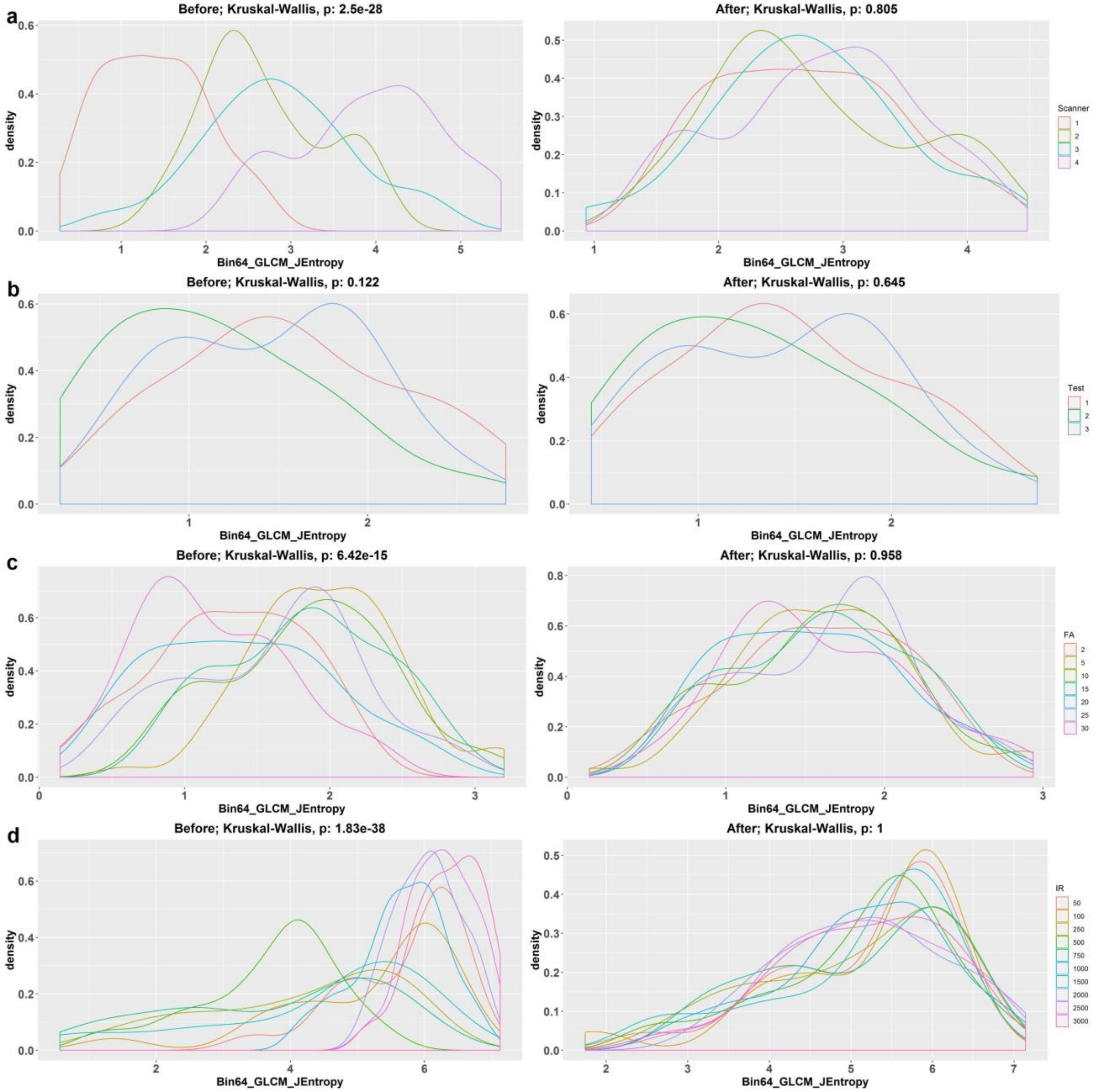

Figure 6. Density plot of Joint Entropy feature from Gray Level Co-occurrence Matrix (GLCM_JEntropy) before and after ComBat harmonization with 64 bins discretizing on: a) four scanners, b) three times test, c) various flip angels, and d) various inversion recovery.

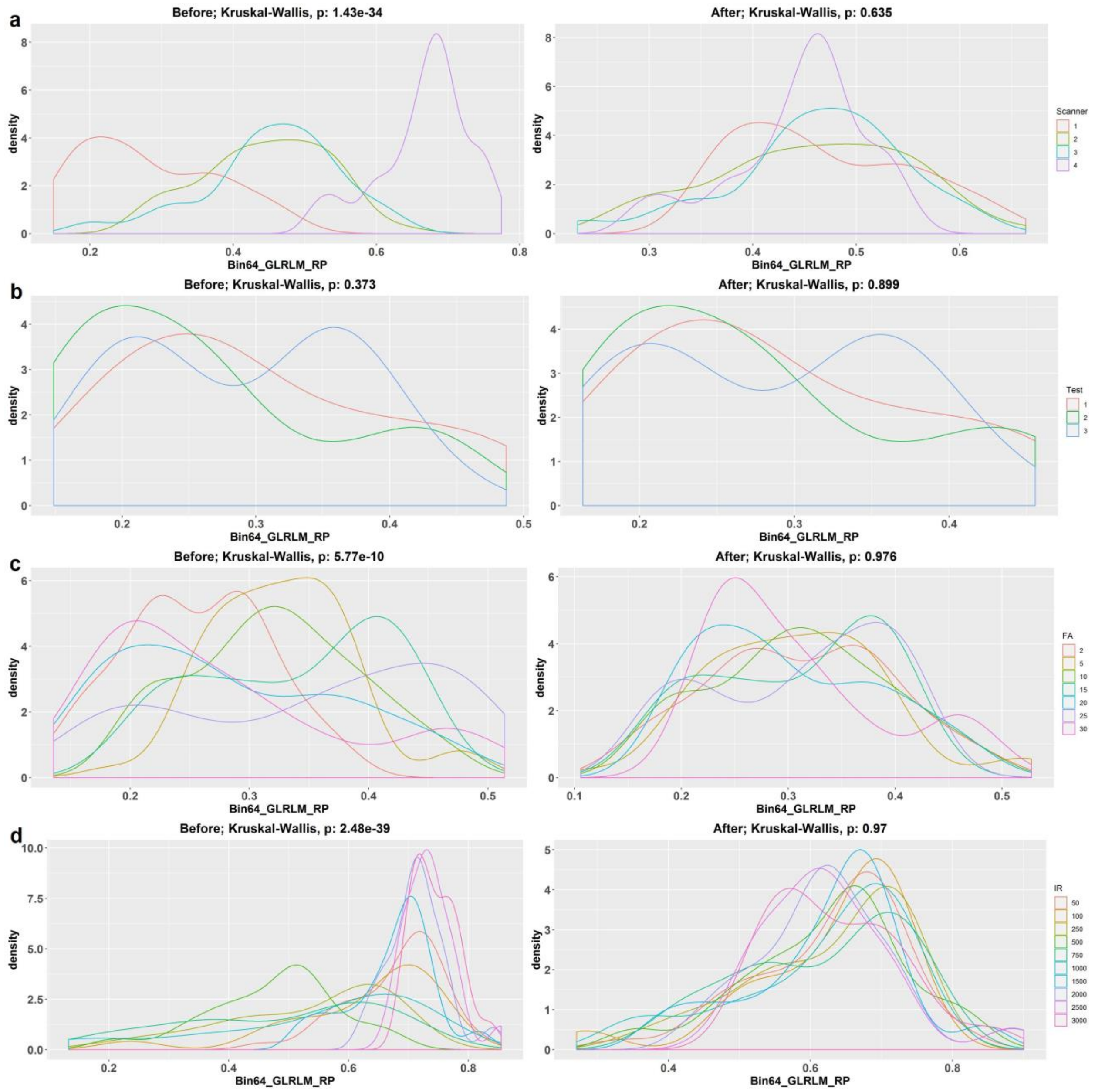

Figure 7. Density plots of Run Percentage feature from Gray Level Run Length Matrix (GLRLM_RP) before and after ComBat harmonization with 64 bins discretizing on: a) four scanners, b) three times test, c) various flip angels, and d) various inversion recovery.

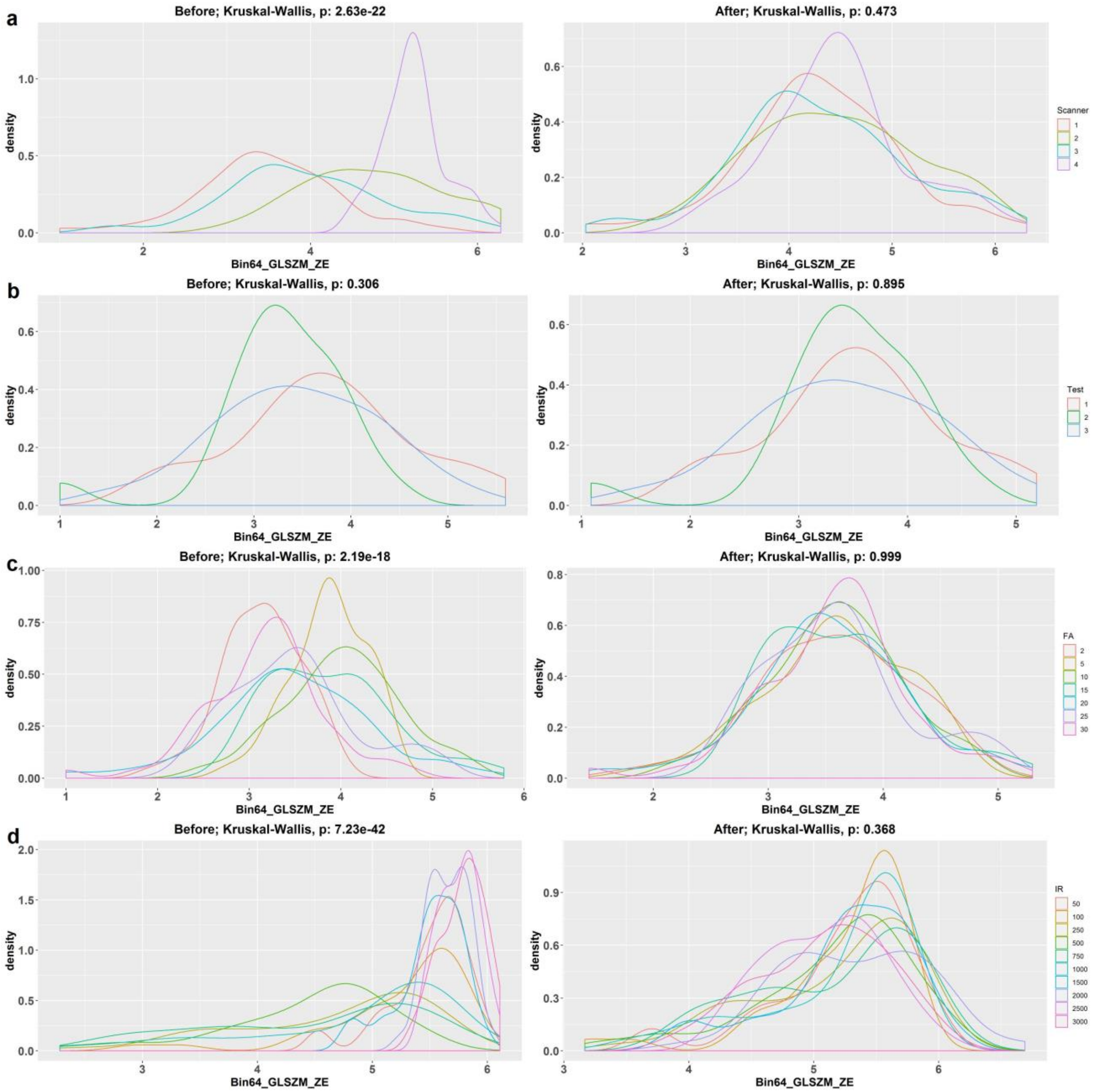

Figure 8. Density plots of Zone Entropy feature from Gray Level Size Zone Matrix (GLSZM_ZE) before and after ComBat harmonization with 64 bins discretization on: a) four scanners, b) three times test, c) various flip angels, and d) various inversion recovery.

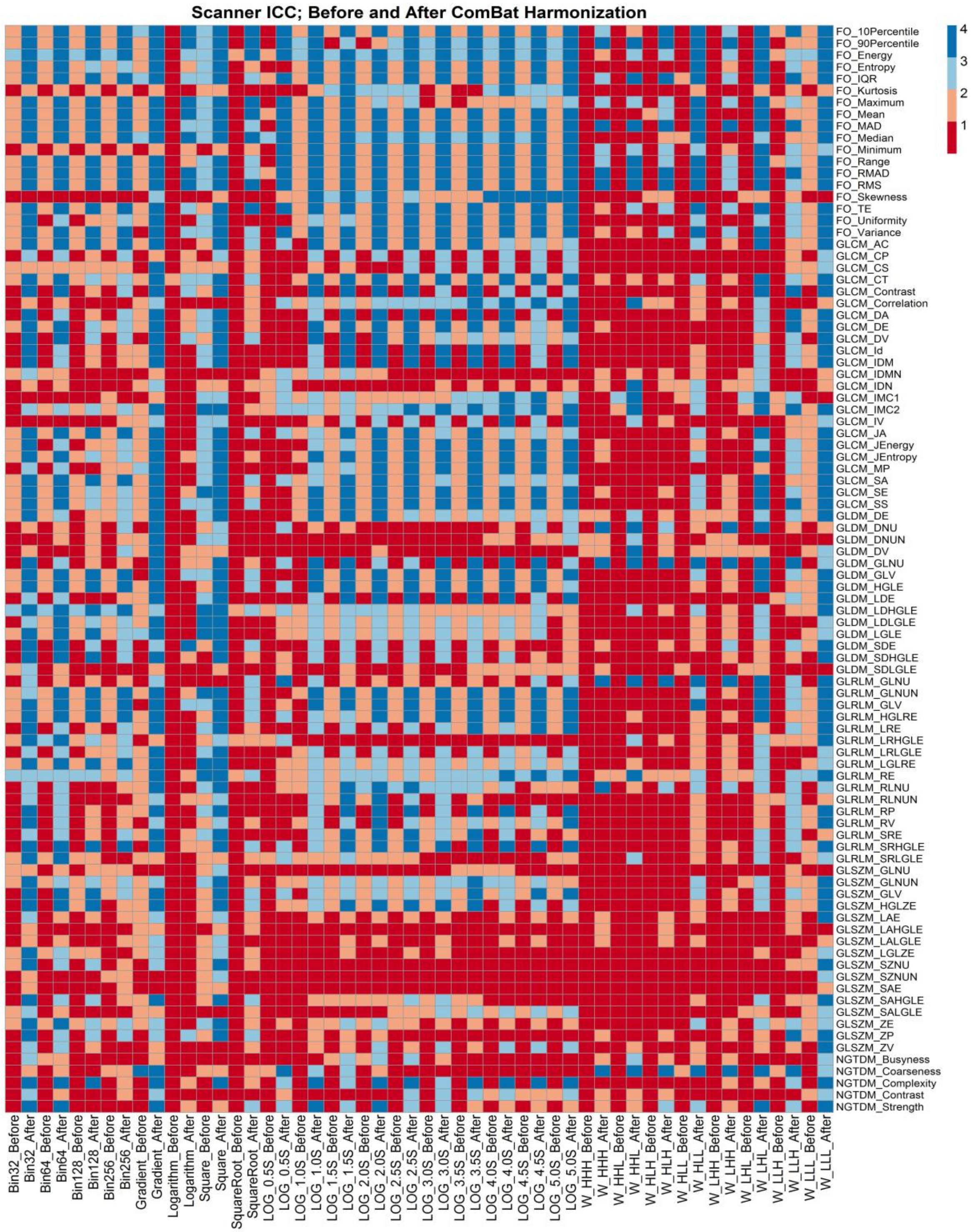

Figure 9. ICC group of radiomic features with different image pre-processing over various scanners. The ICC value is categorized into 4 groups; 1) ICC<50% (low robustness, dark red), 2) 50%≤CC<75% (medium robustness, light red), 3) 75%<ICC<90% (qualified robustness, light blue), 4) ICC>90% (high robustness, dark blue).

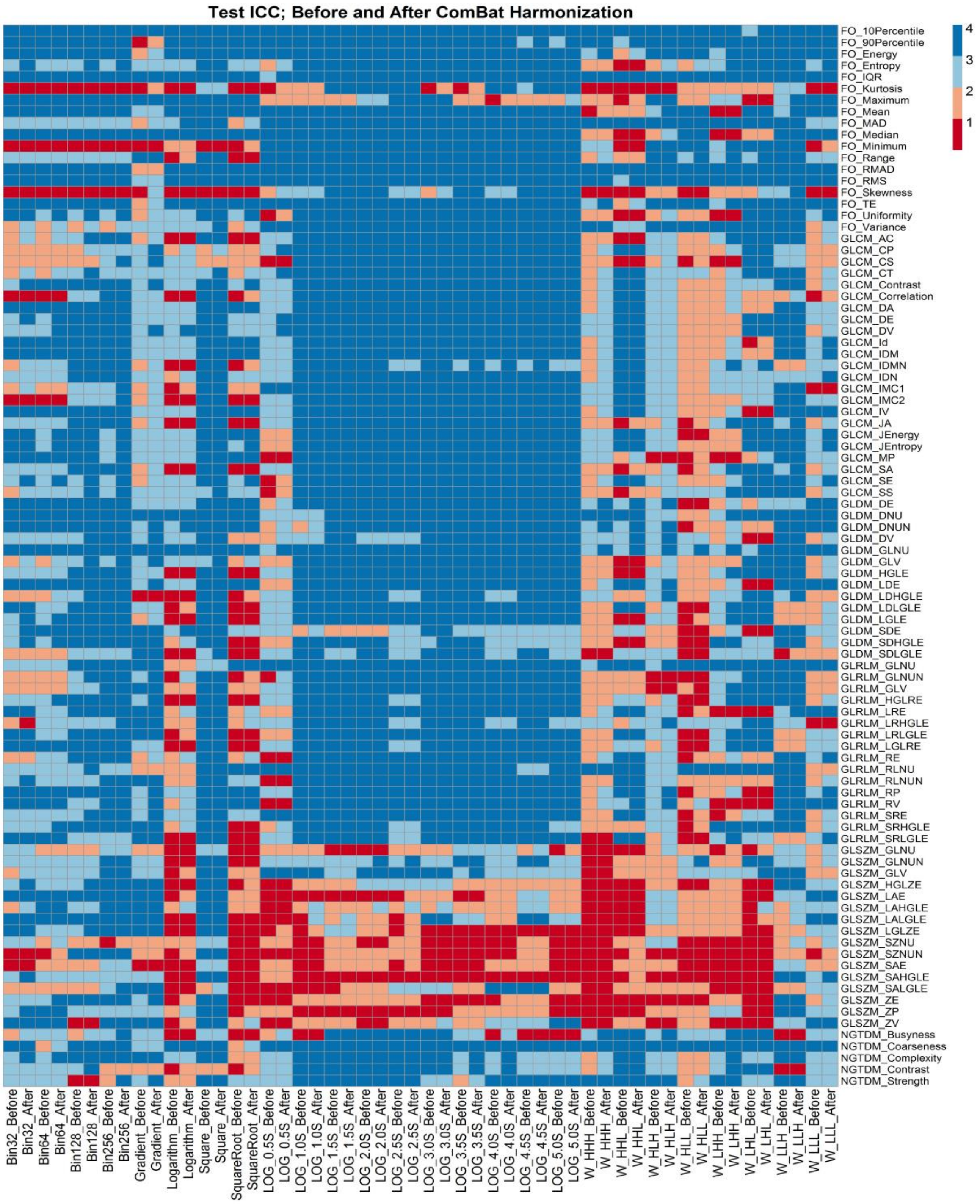

Figure 10. ICC group of radiomic features with different image pre-processing techniques over three-time tests. ICC value is categorized into 4 groups; 1) ICC<50% (low robustness, dark red), 2) 50%≤CC<75% (medium robustness, light red), 3) 75%<ICC<90% (qualified robustness, light blue), 4) ICC>90% (high robustness, dark blue).

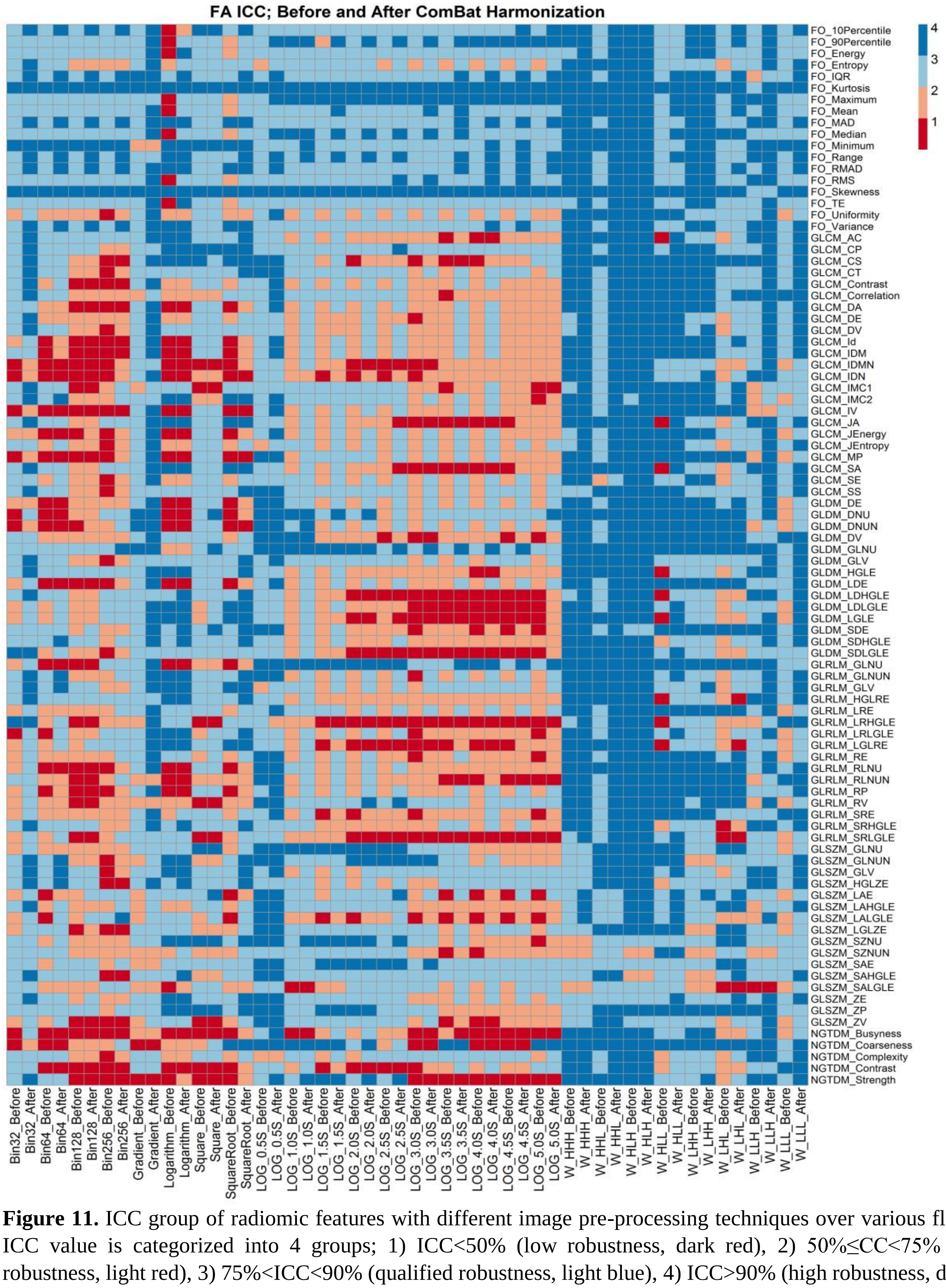

Figure 11. ICC group of radiomic features with different image pre-processing techniques over various flip angles. ICC value is categorized into 4 groups; 1) ICC<50% (low robustness, dark red), 2) 50%≤CC<75% (medium robustness, light red), 3) 75%<ICC<90% (qualified robustness, light blue), 4) ICC>90% (high robustness, dark blue).

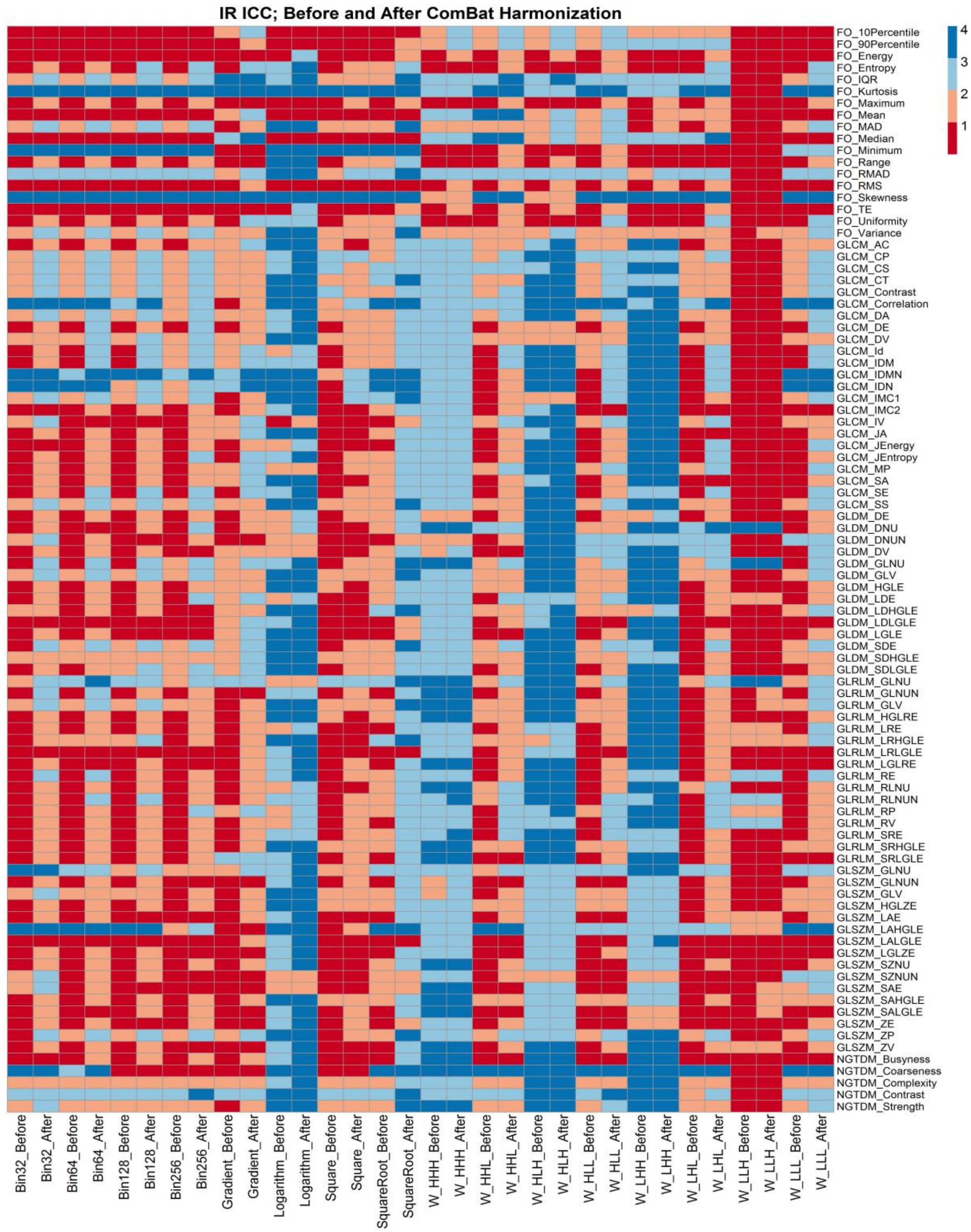

Figure 12. ICC group of radiomic features with different image pre-processing techniques over various inversion recoveries. ICC value is categorized into 4 groups; 1) ICC<50% (low robustness, dark red), 2) 50%≤CC<75% (medium robustness, light red), 3) 75%<ICC<90% (qualified robustness, light blue), 4) ICC>90% (high robustness, dark blue).
